# Supplementary material for: Learning curve and surgical outcome of robotic assisted colorectal surgery with ERAS program
Source: Sci Rep. 2022 Nov 29;12:20566. doi: 10.1038/s41598-022-24665-w (PMC9709162; doi:10.1038/s41598-022-24665-w)
Supplement: Supplementary file 2 — Supplementary Information 2. [file 41598_2022_24665_MOESM2_ESM.zip › RAL Raw data-Ñ[▒K/098/OP_ñw╝╨░O▒Kñσ.pdf]

正式摘要(電子簽章：000000221419848)

W95 / W95

臺中榮民總醫院

# Operation Note

[redacted]

- 手術日期 : 2021/07/15
- 手術醫師 : VS 蔣鋒帆 , CR 林陳彥 , R 郭連雄
- Anesthesia : GE
- Aseptic agents : Betadine Aqua , Zephnan

## Diagnosis

- Pre-operative diagnosis : GIST of rectum with vaginal invasion s/p Gilvec
- Post-operative diagnosis : s/p wedge resection over rectum and vaginal tumor + loop ileostomy formation

## Surgical Indication

- For removing the rectal tumor

## Operation

- 1. Robot (Xi) assisted wedge resection of the rectum and the vagina + loop ileostomy
  2. Intraoperative colonoscopy : (-).
  3. High ligation (-). IMA was not approached.
  4. Take down from the splenic flexure : (-)
  5. No Robotic endoGIA was applied.
  6. Proximal cut end or distal cut end : Could not be evaluated
  7. Loop ileostomy was created over the RLQ of the 8mm trochar site
  8. A 12 Fr. penrose drainage tube was inserted from LLQ of abdomen into pelvic area
  9. ADHESION BARRIER FILM was placed below the fascia of the central umbilicus wound

## Finding

- 1. Tumor could be palpated over the right lateral and posterior site of the lower rectum. Finger tip could reach easily.
  2. Severely adhesion was presented especially over T-colo and the right lateral abdominal wall. Lysis was performed first.
  3. Wedge resection was done over the lower rectum. Nearby vagina was also partially resected due to possibly tumor invasion. The defect area were both sutured with 3-O V-Loc.

## Procedure

- 1. General endotracheal anesthesia was induced and then the patient was placed in modified Trendelenburg lithotomy position.
  2. Preoperative antibiotics were given. A Foley catheter and nasogastric tube were placed.
  3. The abdomen was prepped and draped in standard sterile fashion.
  4. A small vertical incision was made above the umbilicus and a 8 mm trocar was inserted into the peritoneal cavity by open method. Peumoperitoneum was established with CO2 inflation.
  5. Laparoscopy was inserted. Severe adhesion was noted over the RLQ area. A 8 mm of trochar was inserted over LUQ of abdomen with another 5mm trocar over LLQ of abdomen first, in order to perform adheison lysis.
  6. After the adhesion bands were freed, the remaining 8mm trochars were inserted over RLQ and another 5mm assisted port was also inserted over RLQ of abdomen.
  7. The sigmoid colon was held by grasper and the peritoneum overlying the mesentery was scored

- with electrocautery.
  8. The colon was mobilized from retroperitoneum overlying the sacral promontory and into the pelvis. The incision was extended laterally and medially across the peritoneal reflection.
  9. The mesorectum was then mobilized using sharp dissection until the levator ani muscle was identified.
  10. DRE was performed at the same time to identifed the adequate resection area.
  11. Wedge resection was then performed over lower rectum carefully to remove the tumor. The severely attached vagina was also partially resected in order to remove the entire tumor.
  12. Running sutures with 3-O V-Loc was applied to close the defect of the rectum and the vagina, respectively.
  13. The repaired defect was tested for leaks with irrigation solution and air introduced through the rectum and was found to be patent and intact.
  14. The midline wound was enlarged and the specimen was removed.
  15. A 12 Fr. penrose drainage tube was inserted from LLQ of abdomen into pelvic area.
  16. The desired ileum site (About 15~20cm from IC valve) was selected for further ostomy construction.
  17. The 8 mm trochar wound over the RLQ area was further extended to creat the site for loop ileostomy.
  18. The ileum was retracted from the desired ostomy site. A window was created under the ileum by dividing the mesentery adjacent to the bowel wall with electrical cautery carefully, with a plastic bridge in placed.
  19. The trochar fascia wounds were all then closed with interrupted sutures via 1-O Vicryl.
  20. ADHESION BARRIER FILM was placed below the central abdominal wound.
  21. The extended fascia wound was also closed with interrupted sutures via 1-O Vicryl.
  22. Massive N/S was applied to irrigate the wound.
  23. The wound was further closed with 3-O Vicryl, and the skin was applied with SurgiSeal.
  24. Moist pads were placed and the ileum was opened. Succus and air were aspirated.
  25. The ileostomy was matured with multiple interrupted mucocutaneous suture via 4-0 Monocryl.
  26. The ileostomy was well formed and viable. An ostomy bag was placed
  27. The patient tolerated the procedure well and was brought to the POR in stable condition.
- Estimated blood loss: minimum
- 器械數量: 正確
- 紗布數量: 正確

---

## Image

- PAINT
  - abdomen

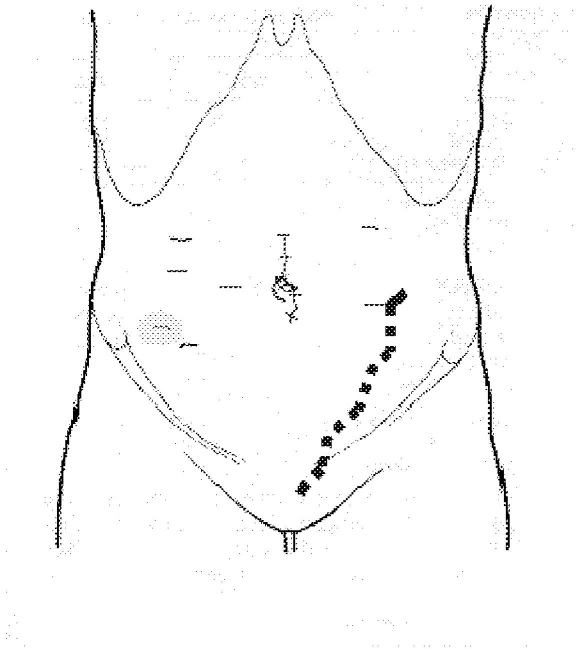

  - colon & rectum

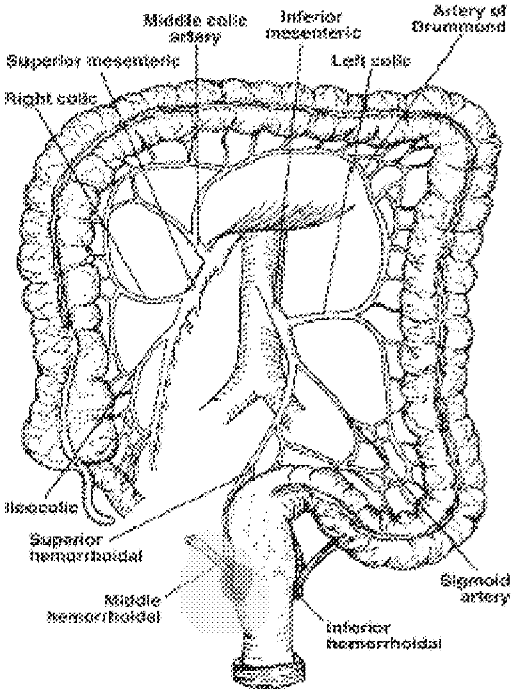


主任 2062F蔣鋒帆 / 醫師 2301H林陳彥

正式摘要(電子簽章：**000000221419848)**

W95 / W95

doctorRank:B
